# The experience of Anxiety for people with Parkinson’s disease

**DOI:** 10.1038/s41531-023-00512-1

**Published:** 2023-05-17

**Authors:** Emma. K. Blundell, Laura. E. Grover, Joshua Stott, Anette Schrag

**Affiliations:** 1 Department of Clinical and Movement Neurosciences, University College London, London, UK; 2 ADAPT Lab, Research Department of Clinical, Educational and Health Psychology, University College London, London, UK

**Keywords:** Parkinson's disease, Parkinson's disease, Parkinson's disease, Neurological manifestations, Quality of life

## Abstract

Anxiety is a common non-motor symptom of Parkinson’s disease (PD) associated with increased disability and reduced quality of life. However, anxiety is poorly understood, underdiagnosed, and undertreated. To date, little research has explored how anxiety is experienced by patients themselves. This study explored the experience of anxiety for people with Parkinson’s (PWP) to inform future research and interventions. Semi-structured interviews with 22 PWP (aged 43-80, 50% female) were conducted and analysed using inductive thematic analysis. Four main themes were extracted: conceptualising anxiety; anxiety and the body; anxiety and social identity; and coping with anxiety. Sub-themes revealed inconsistent perceptions: anxiety was in body and mind, part of disease and human nature, part of self-identity and a threat to it. The symptoms described were diverse. Many perceived their anxiety as more incapacitating than motor symptoms or capable of amplifying them, and described that anxiety restricted their lifestyle. All perceived anxiety as connected to PD, and ultimately persistent: dominant aspirations were coping and acceptance rather than cures, with medications strongly resisted. Findings highlight the complexity and high importance of anxiety for PWP. Implications for therapeutic approaches are discussed.

## Introduction

As a progressive neurological disorder, Parkinson’s disease (PD) presents many physical and psychological challenges to diagnosed individuals and carers^1^. Emotional difficulties such as anxiety occur more frequently in people with Parkinson’s (PWP) than in the general population^2^, but it is unclear how much anxiety is a symptom of PD, a reaction to it, related to medication effects, or caused by external factors unrelated to PD^3^. To date, few pharmacological or psychological treatments have been clearly demonstrated as effective in trials^4–6^ and there is little understanding of how anxiety is experienced by PWP themselves. Greater understanding of patients’ experiences of anxiety is important, as anxiety is one of the strongest predictors of quality of life in PD, can be more challenging to live with than motor symptoms^7,8^, and has been identified as a research priority by PWP themselves^9^.

Anxiety can be defined as an excessive feeling of worry or unease, indicated by an overestimation of threat and underestimation of ability to cope in everyday life^10^. Disorders are typically classified according to the *Diagnostic and Statistical Manual of Mental Disorders (DSM-V)*^11^ and characterised by physical, cognitive, and behavioural components, such as trembling, catastrophising, and avoidance behaviour. Generalised anxiety disorder, social anxiety and a category named “anxiety not otherwise specified” are the most observed in PD^12^. However, one review showed 31% of PWP fulfilled criteria for multiple anxiety disorders, whilst another showed 11.4% had clinically relevant symptoms without meeting any threshold^2,13^. Studies show that anxiety is frequently missed in consultations^14,15^. Detection is complicated by overlapping symptoms such as tremor, fatigue, and loss of appetite, as well as PD-specific symptoms such as increased freezing of gait^16,17^. Whilst detection has been improved by the development of disease-specific screening measures^15^, the *DSM* criteria have yet to be verified for use in PD, which makes accurate classification elusive^12^. It remains unclear whether there is a subsyndromal form of anxiety unique to the disease.

The pathogenesis of anxiety in PD is uncertain. The traditional dopaminergic model of PD suggests underlying dopaminergic dysfunction and neuroanatomical alterations may impact emotional processing^3^. This is supported by retrospective evidence associating anxiety with disease progression and withdrawal of dopaminergic medication^12^ whilst post-mortem studies have reported degeneration of the amygdala, a key brain structure for fear responses that receives mesolimbic dopaminergic input^18^. However, dopaminergic medication does not alleviate anxiety in the same way as motor deficits^19^. Further, retrospective evidence shows anxiety can predate motor symptoms by two decades, whilst prospective evidence shows that anxiety is higher than in controls in medication-naïve patients at diagnosis^20,21^. It is now considered likely that the neuropathology of PD affects the fear circuit in various ways, and that anxiety may correlate with large-scale network connectivity changes that implicate cognitive changes before the onset of symptoms^22,23^. Early pathophysiological changes to brainstem structures involved in stress responses, including the raphe nucleus, rich in serotonin, and the locus ceruleus, rich in noradrenaline, are two candidate mechanisms^24^. Evidence also suggests anxiety in PD is associated with family history of anxiety and biological sex, which may implicate genetic factors and gonadal hormones^25,26^. The neural substrate of anxiety in PD is thus likely a complex interaction of PD and other neurodegenerative disease pathology, involving multiple neurotransmitters.

Anxiety in PD is likely affected by psychosocial factors. Those important for anxiety in non-PD populations include stressful life events, loneliness, and neuroticism^27^. A recent systematic review identified personality, coping, social support, and illness as psychosocial predictors of anxiety in PD. Avoidant and pessimistic personality types were related to higher anxiety, likely due to less successful coping styles such as an avoidance of negative emotional experiences, whilst social support and sense of identity were found to be protective^28^. These results suggest that psychosocial adjustment to the chronic illness contributes to emotional well-being. Older individuals may also have other sources of anxiety, including health issues, finances, and family, or be more likely to normalise fear and have difficulty recognising anxiety symptoms, which could reduce diagnostic sensitivity^29^. It is thus likely that the development of anxiety in PD is multifactorial and affected by how anxiety is perceived.

Little research has explored how anxiety is perceived by PWP. This is important for detection, prevention, and treatment, as perceptions likely affect help-seeking, communication with health care professionals and the management of anxiety over time. Studies suggest long-term health conditions and old age affect engagement with mental health services^30,31^, likely driven by how difficulties are experienced, how they may be perceived by others, and the perceived appropriateness of support available^32^. It is thus important to be aware of how anxiety is experienced by PWP to generate explanatory models and deliver interventions that address their expectations for treatment.

This study explores the lived experience of anxiety for PWP. To our knowledge, only one qualitative paper has explored the subjective experience of anxiety. Its findings highlighted the importance of anxiety in the subjective experience of PD; however, the study interviewed only six participants, and the causes and symptoms of anxiety described were diverse^33^. Our study uses a bigger sample across a larger age range to ask, “How do PWP perceive their anxiety?” and “How do these perceptions affect the maintenance and management of their anxiety?” The aim was to support investigation and treatment approaches by better understanding this important non-motor symptom of PD.

## Results

### Participant characteristics

Interviews were conducted with 22 PWP aged between 43 and 80 years, mean age = 66, 50% female (see Table 1). All self-reported current anxiety. Seven reported taking anxiolytics. GAD-7 scores measuring anxiety at interview ranged from 0 to 19 (clinical cut-off > 7)^34^, mean score = 6. GAD-7 scores at the time of AND-PD assessments ranged from 1 to 19, mean score = 8. PAS anxiety scores ranged from 2 to 47 (cut-off > 13)^15^, mean score = 18, with ten participants above the cut-off score for anxiety. ACE-III scores ranged from 89 to 100 (cut-off for mild cognitive impairment $\le$ 89)^35^, mean score = 96. Two participants reported diagnosed clinical depression at recruitment.

**Table 1:** Participant characteristics.

Participant characteristics	*N* = 22
Age (years), mean ± SD	66 ± 9.54
Sex	
Female, *n* (%)	11 (50)
Male, *n* (%)	11 (50)
Ethnicity	
White British, *n* (%)	20 (90.9)
Other, *n* (%)	2 (9.1)
Dominant hand	
Left, *n* (%)	3 (13.6)
Right, *n* (%)	19 (86.4)
Marital status	
Married, *n* (%)	16 (72.7)
Widowed, *n* (%)	3 (13.6)
Separated, *n* (%)	1 (4.5)
Single, *n* (%)	2 (9.1)
Living situation	
Living with spouse, *n* (%)	16 (72.7)
Living with family, *n* (%)	2 (9.1)
Living alone, *n* (%)	4 (18.2)
Living in	
City, *n* (%)	4 (18.2)
Major conurbation, *n* (%)	2 (9.1)
Town, *n* (%)	13 (59.1)
Village, *n* (%)	3 (13.6)
Employment Status	
Employed, *n* (%)	3 (13.6)
Self-employed, *n* (%)	1 (4.5)
Unemployed, *n* (%)	2 (9.1)
Retired, *n* (%)	14 (63.6)
Highest Qualification	
Secondary education (eg. GCSE, O-Level, A-Level), n (%)	7 (31.8)
Higher education (eg. University degree, vocational course), n (%)	14 (63.6)
No qualifications, *n* (%)	1 (4.5)
Age leaving education (years), mean ± SD	20 ± 3.58
Duration of Parkinson’s disease (years since diagnosis), mean ± SD	7 ± 8.65
Hoehn and Yahr scale of Parkinson’s progression (scored from 0-5)	
1 (Unilateral involvement only), *n* (%)	7 (31.8)
2 (Bilateral involvement without impairment of balance), *n* (%)	2 (9.1)
3 (Mild to moderate involvement; postural instability but physically independent), *n* (%)	13 (59.1)
Experiencing anxiety currently or experienced anxiety in the past, *n* (%)	22 (100)
Family history of anxiety (yes), *n* (%)	8 (36.4)
Family history of depression (yes), *n* (%)	8 (36.4)
Family history of dementia (yes), *n* (%)	5 (22.7)
Family history of Parkinson’s disease (yes), *n* (%)	4 (18.2)
Long-term mental or physical health condition other than anxiety, *n* (%)	14 (63.6)
GAD-7 anxiety score (scored from 0 to 21), mean ± SD	6 ± 4.71
Anxiolytic medication status (currently taking), *n* (%)	7 (31.8)
GAD-7 anxiety score at interview (scored from 0 to 21), mean ± SD	6 ± 4.71
Participants with complete PAS and ACE-III scores, *n* (%)	18 (81.8)
	***N*** = **18**
PAS anxiety score at AND-PD assessment (scored 0–48), mean ± SD	18 ± 11.58
GAD-7 score at AND-PD assessment (scored from 0–21), mean ± SD	8 ± 5.22
ACE-III score at AND-PD assessment (scored 0–100), mean ± SD	96 ± 3

### Themes

The paper reports four themes: conceptualising anxiety; anxiety and the body; anxiety and social identity; and coping with anxiety. These are introduced by an exemplar quote followed by a presentation of salient sub-themes (see Table 2). Themes should not be regarded as independent; participants expressed contradictory ideas and themes interact. Whilst quantifying language is used to suggest prevalence, such terms are not used to “count” examples but rather give an idea of consistency of topics that were raised spontaneously across interviews. A semi-structured topic guide allowed for topics to be generally consistent. Within each theme, the respondents were compared across biological sex and whether they scored above the cut-off score on the PAS. Cross sub-sample similarities in thematic content dominated and there were no apparent sex differences. Only where areas of difference according to PAS scores were found are they reported.

**Table 2:** Themes identified and their prevalence across participants.

Themes	Sub-themes	*N* = 22	%
Conceptualising anxiety	Fusion from self	14	64
	Defusion from self	16	73
	Alienation from self	7	32
Anxiety and the body	States of the body	20	91
	Changes in the body over time	22	100
	Part of a chronic condition	17	72
Anxiety and social identity	Part of identity	22	100
	Perceptions of others	22	100
Coping with anxiety	Ability to cope	20	90
	Strategies used	21	95
	Professional support	22	100

### Conceptualising anxiety

*“It’s a condition, a state of mind, a state of being”* [P3]

Participants perceived anxiety as a state with an altered sense of self. This was experienced in different ways.

#### Sensory experience

Over half of participants perceived anxiety through sensory terms (“just go completely cold”) which demonstrates a difficulty describing anxiety conceptually (“very difficult to explain”). For some, anxiety was considered beyond conscious awareness:

*“I think to some extent it’s an unconscious thing.”* [P25]

#### External intrusion

In contrast, some perceived the felt experience of anxiety as distanced from the self, as if an alien presence (“nebulous thing”) inhabited their body. Idioms (“headless chicken”) and personification (“other voice”) conveyed how this presence threatened human agency, with the body often described using animal imagery (“meerkat”). This set up a dualistic negotiation between body and mind:

*“the body thinks, OK […] you’re getting yourself into that situation again. So, I’m already alerted to the fact things aren’t right.”* [P25]

A quarter of participants perceived anxiety as an intrusion on the self, conceptualised as a conflict:

*“by creating this metaphor image it actually helps me fight […] I consider it a battle of wills and I intend to win.”* [P5]

#### Depersonalisation

Anxiety was perceived as capable of exerting its own volition over participants, which could result in destabilising experiences:

*“the world sort of inverts, and sort of focuses on you, and you just feel like floating through this sort of scene of stuff going on, feeling really odd.”* [P13]

For some, anxiety led to depersonalisation and loss of self, as if the mind and body became separate:

*“you couldn’t really get out, you couldn’t really go anywhere, because your body was sort of here, but you were elsewhere.”* [P25]

### Anxiety and the body

*“I don’t know if it’s the anxiety or the Parkinson’s, but I feel like I’m shaking on the inside.”* [P18]

All but three participants perceived anxiety as a physical experience. For most, anxiety came in episodes (“waves”) alongside other PD symptoms, whereas for some anxiety was perceived as a reaction to them. Ultimately, most perceived anxiety as part of an incurable, chronic motor disorder.

#### States of the body

Participants perceived anxiety as physical changes to body and brain. Internal experiences were most frequently described, such as shallow breathing, nausea, and heat (“hotness”). Changes to the visible body were perceived as a worsening of PD motor symptoms, including increased tremor and stiffness, as if becoming less human (“like the incredible hulk”). Some perceived increased tremor internally (“shaking on the inside”). Somatic experiences varied in duration but lasted until anxiety had eased. Alterations to the brain were perceived as causing deleterious changes to thought processes, often described through motor terminology (“freezing […] in thought”). Physical experiences of anxiety were often claustrophobic:

*“my brain racing but my body seems to slow down at the same time […] a feeling of wanting to withdraw and not feel trapped.”* [P15]

#### Changes in the body over time

For most, anxiety was a fluctuating presence (“peaks and troughs”) that was sometimes manageable (“a lot of the time you are perfectly OK”) and sometimes not (“other times I just chew away at something in my brain”). Some described anxiety as self-perpetuating (“circle”). The cause of anxiety fluctuations was often unclear (“can’t pinpoint why”) and inconsistent. However, some noticed patterns with fatigue:

*“the fatigue is a bit of a killer, where does that come from? And whether it’s the fatigue that starts off the anxiety? I know that when I’m anxious, fatigue is there.”* [P25]

Fluctuations in anxiety were also related to fluctuations in low mood and depression (“my mood level is always fluctuating”) particularly for participants with higher scores on the PAS, although some found low mood hard to differentiate from anxiety (“hard to tell what is what”). Depression was often perceived as the result of living with anxiety:

*“anxiety stops me doing things […] I am depressed about that.”* [P8]

The perceived relationship between anxiety and PD medication was inconsistent. Some considered anxiety might be caused by medication (“side effect”), others believed medication could prevent its onset (“even though it wasn’t due I just took my next dose”) and two stated that any efficacy might be psychological. Most perceived PD medication was not responsible:

*“I don’t think it’s to do with the medication, I think it’s to do with the neurological problems that I’ve got.”* [P23]

#### Part of a chronic condition

Anxiety was perceived as one of the earliest and most incapacitating PD symptoms (“I would put up with some of the motor disability if it replaced the anxiety”) that will never cease (“whilst it does go up and down, I think it will affect me in future”). Some wondered whether anxiety was a risk factor for PD:

*“I’ve always been a stressy person […] it does make me wonder whether being that type of person makes me more prone to getting Parkinson’s.”* [P7]

Most thought anxiety must be implicated in disease pathology:

*“It’s definitely got worse with Parkinson’s, so I’m sure it’s to do with the illness itself.”* [P2]

Some alternatively suggested anxiety was a reaction to having PD and uncertainty around disease progression (“paranoia about dying”). Taking medication for anxiety was resisted, out of concern drugs might further overwhelm the body (“too many drugs in the system”) or signal deterioration (“If I’m taking more medication […] I must be getting worse”). All thought anxiety was related to PD in some way, and thus ultimately persistent:

*“I just feel it will always be with me.”* [P1]

### Anxiety and social identity

*“It’s as if I’m two people. I’ve got my anxious side and then the side I’m presenting to the public […] I switch from one to the other.”* [P7]

Participants stated that anxiety had changed their identity (“robbed of my own resilience”) considered here as how they viewed themselves compared to other people. Some considered anxiety a personal weakness (“I didn’t want them to think I was anything but Superman”) associated with stigma. This had led to a sense of isolation (“go back into a bit of a shell”). Social connection was perceived as an important source of support.

#### Part of identity

Participants perceived that anxiety was related to various personal life events, which changed their identity over time. For some, anxiety was perceived as innate (“the way I’m made”) whereas for others, a transformation had been radical (“gone from highly resilient”). All felt less able to cope with life due to anxiety:

*“the feeling that you’re not very good, you can’t cope, you can’t do things […] it’s all tied up with your sense of yourself as worthless.”* [P15]

Identity was perceived to be affected (“like kryptonite”) by symptoms of anxiety that compromised social roles (“led to early retirement”). Participants described a need to plan situations in advance to manage anxiety. Anxiety also compromised communication skills, including difficulty interpreting others (“coping with all the conversations”) and speaking (“higher in pitch, maybe a more strained voice”). Participants thus felt less sure of themselves around others:

*“I was quite eloquent and would talk well and react quickly and be funny, and I’ve now noticed because I’m not like that, and you notice. I dunno, I think you get anxious about knowing that people are going to spot that.”* [P21]

#### Perceptions of others

Participants described anxiety about social evaluation (“fear that I’ll be rejected”) that necessitated hiding anxiety from others (“managing…the outward persona”). Perceived stigma was more commonly described among participants who scored below the PAS anxiety threshold:

*“It feels like a private, almost a private thing, which is silly really, because I’d happily talk about my physical health to people.”* [P16]

Participants also concealed anxiety to not trouble others (“burden them”) or admit they couldn’t cope with PD, as this might reduce their independence:

*“I certainly wouldn’t want to be in a position where I’m being looked after.”* [P24]

However, nearly all participants stated others would not consider them anxious, even if told. Others either misunderstood (“they seem to think it’s sort of something funny”), lacked empathy (“I don’t think he really gets it”) or lacked awareness:

*“They probably just think because I’ve got Parkinson’s then the shaking comes with it.”* [P11]

Around half stated they would share their anxiety with the right people, particularly other PWP:

*“They’re all diagnosed quite a long way ahead of me […] they’ve normally got stories that oh yes, that happened to me, and I did this, which is really helpful.”* [P4]

### Coping with anxiety

*“I know it’s not a constant thing and I know there are ways to understand it, control it, manage it.”* [P20]

Participants’ views on whether anxiety could be managed differed. For some, avoidance strategies had resulted in a restricted lifestyle. Most had not sought help from healthcare professionals. There were differing views on psychological interventions, but most perceived their utility if in a suitable format. The dominant aspiration was coping.

#### Ability to cope

Two-thirds of participants perceived they could cope with anxiety. Participants who scored below the PAS anxiety threshold more commonly described using a stoic attitude (“mind over matter”) to defy anxiety. Other participants aspired to accept it:

*“I’d like to find a place where it’s there […] not necessarily control it, but understand it for what it is, you know, accept it and live with it kind of thing.”* [P20]

A third felt they had no control over anxiety:

*“I don’t think I have any control over it […] It seems to me it just happens, that’s what it feels like. It doesn’t have a logical explanation to it.”* [P17]

A few conveyed that anxiety was so uncontrollable, it had contributed to suicidal ideation:

*“I’ve had suicidal thoughts, thoughts that I’m a failure, thoughts that I’ve let my family down, uhm, thoughts that I should be able to get out of this by myself, and clearly, I can’t get out of it by myself.”* [P18]

#### Strategies used

Cognitive strategies described included distraction, rationalising (“I’ve done this before I can do it again”) and mindfulness:

*“you just try and live in the present all of the time, which is a very Buddhist way of thinking as well […] that is the way I switch my mind.”* [P7]

Behavioural strategies included prioritising enjoyable activities, learning new skills to increase confidence, social interaction, and exercise due to perceived effects on the brain (“other parts of the brain take over”). Some journaled to monitor patterns:

*“I could see my score, how I was feeling today, and I used it to monitor that every week, every month, so I could see how I was doing and that is such a useful tool.”* [P3]

Avoidance was often used when other strategies were inadequate (“I prefer to isolate myself”). Social situations that carried a risk of negative evaluation (“fear of failure”) were most often avoided. This had a deleterious impact on lifestyle:

*“It ruins a lot of things because I can look forward to something and then get there and it’s so awful, I have to leave […] it limits what I can do.”* [P10]

#### Professional support

Most participants had not sought support from healthcare services, and many had not been offered support (“they’ve not mentioned it”):

*“mental wellbeing is just as important as some of the physical symptoms that you’re dealing with. And I think when the time is so pressed […] it’s too easy to concentrate on the physical problems.”* [P19]

Participants felt anxiety was misunderstood (“they still do not understand anxiety”) by professionals:

*“I wish the wider healthcare world would view it as something that is part of the condition, rather than a consequence of having to deal with it.”* [P20]

Participants also described lacking motivation to seek help (“I’ve got the paperwork, pamphlets, but no, I’ve not contacted anybody”) partly because they felt more comfortable discussing physical symptoms:

*“I have a tendency to pretend everything’s alright when it isn’t […] I’ll talk about things that are kind of obvious, like you know, the obvious physical things.”* [P16]

Psychological interventions were the preferred from of treatment, but some participants evaluated their experiences as negative. Reasons included: the therapist (“didn’t trust her”), homework (“couldn’t seem to take reason from it”), lack of personalisation (“generic”) and lack of PD-relevant content (“if it’s a chemical thing […] how can CBT have any effect?”). Other participants evaluated their experiences as positive:

*“CBT I didn’t find that useful for me, for my personality type […] counselling stroke supervision on a much broader range of things, that was a real life saver for me when things were really bad.”* [P13]

All but two participants were resistant to medication (“unless it becomes essential”) due to a perceived risk of side effects or withdrawal effects (“you can’t just come off it”). Many had considered alternative therapies, including hypnotherapy, massage, and magic mushrooms (“it’s supposed to form or project neural pathways”). These were preferable because they were natural:

*“I’m quite happy to have a go at natural products rather than fabricated medicines.”* [P7]

Whilst some wished anxiety would disappear (“disappear completely”) nearly all thought anxiety was inevitable and aspired to self-management:

*“Take a little bit of the meerkat away […] I wouldn’t want it to be taken away totally because I don’t think that’s natural. To me, there’s a reason why I do this, why my body does this.”* [P1]

## Discussion

We identified a wide range of experiences and perceptions of anxiety in PWP, arranged into four themes: conceptualising anxiety; anxiety and the body; anxiety and social identity; and coping with anxiety. A tension was found between anxiety and the self, communicated through diverse, sometimes conflicting cognitions: anxiety was in body and mind; part of their identity and part of the biological illness of PD; and subject to frequent fluctuations. Participants highlighted the incapacitating impact of anxiety on health, wellbeing, and identity, but felt anxiety was ultimately persistent and so aspired to acceptance.

Anxiety was perceived as a state of mind affecting the self. Some conceptualised this as a sensory feeling; for others anxiety was an intrusion, conceptualised as a voice; a few experienced feelings of depersonalisation. These distinct perceptions of anxiety as either part of the self (cognitive fusion) or as thoughts detached from the self (defusion) are important, as cognitive fusion may lead to psychological inflexibility and avoidance^36,37^. All participants described avoiding situations where they felt unable to manage internal experiences. This suggests participants often found it difficult to cognitively defuse and prevent anxiety becoming overwhelming.

Anxiety was perceived as a physical experience connected to their motor disorder with a reciprocal increase in co-existing symptoms. Anxiety often co-occurs with PD symptoms including sleep difficulties, motor skills, and depression^17,38^. However, participants with lower PAS scores were less likely to describe their anxiety as connected to low mood, which may lead to lower detection rates in those with anxiety without depression. Anxiety in PD may also fluctuate like motor symptoms^39^. Participants used motor terminology to describe anxiety, such as “shaking on the inside”, which would suggest a common phenomenological experience with the subjective complaint of “inner tremor” relating to dopamine deficiency^40^. The confluence of anxiety with motor symptoms suggests that anxiety may be related to off-periods between doses of dopaminergic medication. Research shows non-motor fluctuations may not always occur with motor off-periods, which would explain why the relationship was not perceived by participants^41^. It is possible that increased tremor or stiffness triggered by anxiety is associated with the physical presentation in non-PD populations^10^, or that anxiety may lead to an increased overall perception of physical symptoms. Recent research suggests interoceptive deficits in PD. Interoception is the perception of physical sensations that represent the physiological states of the body, including those described by participants^42,43^. Emotional processing and interoception involve overlapping areas of the brain, including the amygdala^44^, and enhanced interoceptive processing has been related to anxiety in the general population^45^. Early research on the neural mechanisms of anxiety in PD suggests that cingulate cortex alterations, associated with interoception deficit, could be a key point of anxiety pathophysiology and treatment^24,25^.

Anxiety was perceived as affecting social identity. For some, anxiety was connected to receiving their PD diagnosis, which suggests anxiety is associated with adjustment to chronic illness^46^. Many participants thought anxiety would be misunderstood as a sign they were not coping with their diagnosis. Studies show that individuals strive to behave according to the norms of their culture and within Western culture, value is placed on being an independent, autonomous entity^47^. This may partly explain why PWP tried to conceal any signs that they may not be coping, were changed from how they were before, or may need to be cared for by others. Perceived social stigma also contributed to anxiety being concealed. As anxiety was perceived to worsen socially important symptoms of PD, including speech problems, many avoided social situations in which it may become apparent. Importantly, avoidance perpetuates anxiety as it minimises opportunities to disconfirm perceived threats; it also may lead to social network attrition or loneliness, which could negatively impact health and well-being over time^48^ and lead to overall worse outcomes.

Participants described a variety of coping strategies. The use of these depended on how anxiety was conceptualised. Some tried to suppress or ‘fight’ internal experiences of anxiety, regarding it as a personal weakness that threatened identity; others tried mindfulness to try and ‘accept’ that experiences were transient. For participants who connected anxiety to somatic sensations, many used behavioural strategies such as exercise and massage. This supports research showing that both alleviate Parkinsonian symptoms and enhance well-being in PD^49,50^. Those who perceived anxiety as affecting social identity found social connection valuable, particularly with other PWP. Indeed, studies of those with long-term health conditions show social networks can benefit wellbeing even if made up of ‘weak’ ties outside of family and friends^51^. However, one participant described resistance to formal support groups with PWP in case they met others in a worse physical condition than themselves, which they perceived would heighten anxiety about disease progression. More research is needed on how social support could decrease self-stigma and facilitate coping.

Few had spoken about anxiety with professionals. Studies show that anxiety is often missed by consultants^14^, and participants perceived physical symptoms were often prioritised in consultations. All but one would prefer psychological therapies over medication for anxiety, but few had been offered these. Older adults are more likely to be given medication for mental health problems and less likely to be offered psychological therapies than younger adults, despite evidence talking therapies may work better in such populations^52,53^. The most widely tested psychological intervention in PD is CBT. Traditional second-wave CBT supposes emotional problems are caused and maintained by unhelpful thoughts and behaviours, that are monitored in therapy by gathering evidence to test appraisals^54^. A recent meta-analysis found CBT to be efficacious in reducing anxiety in PD, although the seven included studies varied widely as regards the duration, intensity, and modality used^6^. However, the change-oriented approach of standard CBT may be in conceptual conflict with our findings. Not all participants had experience of CBT and it may be that some received an integrative approach to psychological intervention; however, participants often described their experience of interventions as generic and inapplicable, as they did not account for the perception that their anxiety was part of an incurable condition and not just a reaction to it. Further trials of CBT protocols adapted for PD-specific factors like falls, freezing, shame, and comorbid neuropsychiatric and cognitive symptoms are warranted^5^.

Participants perceived anxiety as ultimately persistent. Many prioritised value-based activities, such as hobbies, exercise, and social interaction, to help minimise its impact; some found mindfulness helpful to change their relationship with anxious thoughts. The dominant aspiration was acceptance rather than cure. This suggests third-wave behavioural therapies, such as acceptance and commitment therapy (ACT) and mindfulness-based therapy (MBT) may be beneficial^55,56^. These approaches build on second-wave CBT by redirecting treatment from testing and changing thoughts towards supporting psychological flexibility and identifying ways to live in accordance with one’s values, despite adversity. Future research could test the efficacy of such therapeutic approaches in PD.

This study has several strengths. To our knowledge, this is one of the first studies to explore anxiety from the perspective of PWP. The sample was purposively sampled for equal numbers of men and women, in line with WHO guidance, and large enough for robust thematic analysis. Our topic guide was informed by PPI feedback and trialled with nurses to minimise researcher influence. An inductive approach to analysis was used to resist premature application of theory based on clinical assumptions. We recruited individuals based on self-report of anxiety thereby including those who may not have been detected by traditional screening methods, as suggested by the relatively low GAD-7 scores, but for whom anxiety was an important part of their subjective experience of PD. Of note, mean scores on the disease-specific PAS were increased but mean GAD-7 scores measured at the same time as the PAS were relatively low. The generic GAD-7 may thus not be a sensitive measure for all aspects of anxiety in PD. Further, we found that participants who scored below the PAS threshold were more likely to perceive stigma around anxiety and adopt a stoic attitude, which suggests such individuals may underreport or conceal symptoms.

There are some limitations. A more diverse sample may have enriched our interpretations including in different cultures and in individuals with severe functional disability. Transcribed interviews were read only by EB, who has no formal training in clinical psychology, although final themes were discussed with author JS, an experienced consultant clinical psychologist. Participants differed in how long they had been experiencing anxiety and interviews may be affected by recall bias. We measured anxiety levels at the time of interview using the GAD-7, but the time interval between interviews and the PAS means results may not fully reflect levels of anxiety in the sample.

Overall, anxiety was conveyed as frequent, debilitating, and often as incapacitating as motor symptoms. Experiences of anxiety were perceived to alter sense of self, body, and social role. The relationship with other PD symptoms and antiparkinsonian medication needs to be carefully explored and communicated to patients, whilst testing should take place periodically to enhance detection, as patients may not seek help. However, patients may resist additional medication or regard a referral to a psychiatrist as an invalidation of symptoms they perceive are part of PD. The findings thus suggest a purely biomedical interpretation of anxiety as brain disease to be targeted with medication would overlook the social, psychological, and behavioural dimensions of illness, and be disjointed from treatment aspirations. Future treatment trials should test formulation-based therapies that include the patient’s explanatory model for their anxiety. Future research should address both the neurobiological and psychosocial mechanisms of anxiety in PD, to better understand how anxiety relates to physical experiences and how it can best be managed.

## Methods

### Design

This qualitative study is part of a larger longitudinal study on the mechanisms of anxiety in PD (AND-PD). We used in-depth semi-structured interviews with PWP conducted between March and April 2022. Qualitative methods were chosen to provide an in-depth, contextualised understanding of individual experiences^57^. An inductive approach to thematic analysis (TA) was chosen as the method of analysis.

### Participant recruitment

Participants were eligible if in the UK, aged between 18 and 89 years, had a confirmed diagnosis of PD, and self-reported anxiety. Anxiety was not defined in advance of interviews, as we were interested in how participants perceived anxiety and did not wish to lead them in their responses. Taking a self-report approach for anxiety meant we avoided discounting the experiences of those experiencing anxiety who fell below clinically significant thresholds. We used a maximum variation strategy and recruited across all disease stages and subgroups. The severity of PD was assessed using Hoehn & Yahr staging (from 1-5; stage 5 as most severely affected)^58^.

Recruitment took place through various channels, including neurology clinics, publicity by the charity Parkinson’s UK, a patient and public involvement (PPI) session, and referral by other participants following their interview. 18 of our participants were also participating in other parts of the AND-PD study. A sampling matrix ensured our final sample was divided equally by sex to ensure adequate representation of females, following recent guidance from the World Health Organisation and Parkinson’s Foundation^59^. Recruitment was ceased at 22 interviews (males = 11) to provide a manageable sample size large enough for adequate comparison between sexes^60^. This sample size is larger than generally considered necessary for scrutinising common perceptions. However, it optimised our ability to collect rich data from different perspectives and is comparable to similar studies^61,62^.

### Data collection

Diligence was taken to ensure interviews were conducted according to participant preferences. All elected to have remote interviews without carers present, so care was taken to ensure the researcher was in a quiet, private place throughout to reduce any anxieties and potential power imbalance. Semi-structured interviews were conducted using video conferencing software. The interview focused on the symptoms, triggers, consequences, and treatment of anxiety (Supplementary File 1). Whilst the topic guide was used to structure interview questions, there was flexibility to follow what each participant discussed in response. Prompts were used to elucidate further detail, such as “could you please tell me a bit more about that.” All interviews thus took a slightly different route from the questions as a starting point, in response to what participants perceived was important for describing their experience of anxiety. No topics were introduced by the interviewer outside of the topic guide, to ensure general consistency. All interviews were conducted by EB. The participants were not known to the interviewer or other authors before recruitment and trust was built by establishing rapport with the interviewer through email and telephone prior to interview. Interviews lasted on average 46 min and with prior consent, were audio-recorded and transcribed. On the day of the interview, all 22 participants reported demographic information and completed the Generalised Anxiety Disorder Scale-7 (GAD-7; maximum score 21, with higher scores indicating higher anxiety)^63^ to assess general anxiety levels. For participants recruited to the larger AND-PD study, anxiety was measured in separate assessments by the GAD-7 and the Parkinson’s Anxiety Scale (PAS)^15^ (maximum score 48, with higher scores indicating greater anxiety). Cognitive impairment was measured by the Addenbrooke’s Cognitive Examination (ACE-III)^35^ (maximum score 100, with higher scores indicating better cognitive performance). The median time interval to interview was 65 days (IQR = 43–78) for the anxiety assessments and 68 days for the cognitive assessment (IQR = 20–90). The 3 participants not recruited to the AND-PD study do not have PAS or ACE-III scores.

### Analysis

Interviews were transcribed verbatim by EB and proofread for accuracy in accordance with guidance from transcription protocols^64^. Data analysis was conducted using NVivo 12 to enable the systematic organisation of codes and themes.

We drew on Braun and Clarke’s six-stage methodology of reflexive inductive TA^65^ underpinned by a critical realist epistemological perspective. This assumes a largely unproblematic relationship between the real world and language, whilst accepting that our understanding of the world is a construction created from our own perceptions^66^. The first stage of reflexive TA is data familiarisation, whereby transcripts were read in different contexts to reduce any effect of mood on the reviewing of the data. Transcripts were then systematically coded to create around 90 codes, but many captured micro-differences, so consequent consolidation reduced this to a workable number. Codes were then formed into potential patterns of meaning that became themes that addressed the research questions using an iterative approach through discussion between EB, JS and AS. Themes were not formed as topic summaries in response to individual interview questions; each aimed to capture a distinct, coherent, internally consistent account of the dataset. An iterative process of analysis clarified these into four themes that were then defined and named. During the last stage of analysis, a report was written using textual extracts, during which codes and themes were refined as necessary. We did not assess interrater reliability as advocated within codebook or coding reliability approaches^67^. Our reflexive approach regards the researcher’s subjectivity as the sculptor of knowledge produced, rather than a threat to its credibility^68^.

### Reflexive statement

Researcher influence in QA is unintentional but inevitable. The researcher (EB) is a white British female in her late twenties. At the outset of the study, she had training in qualitative research in the context of an MSc in Psychological Sciences but no formal training in clinical psychology. To maximise rigour, a reflexive journal was used throughout the study for self-reflection and the ‘bracketing’ of potential biases^69^.

### Ethics and patient and public involvement

This study is part of the AND-PD Project approved by University College London Research Ethics Committee (REC21/YH/0016). We used a PPI group and pilot interviews with NHS nurses to gather feedback on research conduct. Interested volunteers for the study were provided with an information sheet and gave informed written e-consent prior to interview. No incentive was offered for participation. If any suicidal ideation was mentioned in the interviews, a further safety assessment was conducted.

### Reporting summary

Further information on research design is available in the Nature Research Reporting Summary linked to this article.

## Supplementary information

Supplementary File 1

Reporting Summary

## Data Availability

The data generated and analysed during the current study are not available as individual privacy could be compromised.
