# Supplementary material for: The experience of Anxiety for people with Parkinson’s disease
Source: NPJ Parkinsons Dis. 2023 May 17;9:75. doi: 10.1038/s41531-023-00512-1 (PMC10192312; doi:10.1038/s41531-023-00512-1)
Supplement: Supplementary file 1 [file 41531_2023_512_MOESM1_ESM.pdf]

Supplementary File 1

Interview topic guide

**Interview questions**
1. What words or images come to mind when you think of anxiety?
2. What symptoms have you experienced that you think are associated with your anxiety?
3. What term or language do you prefer when referring to anxiety?
4. What do you believe may have caused your anxiety?
5. Are there any situations or triggers that make your anxiety worse?
6. Are there any situations or triggers that make your anxiety better?
7. Do you think your anxiety is linked to your other Parkinson's symptoms or Parkinson's medication?
8. Has your anxiety ever been accompanied by feelings of depression?
9. When did you first notice feeling anxious?
10. In what ways has your experience of anxiety changed over time?
11. Would you describe the anxiety as chronic or acute or relapse-remitting?
12. How does anxiety affect your life now?
13. Does anxiety affect you physically?
14. Does anxiety affect your ability to concentrate or pay attention?
15. Does anxiety affect how you think or feel about yourself?
16. How do others react to your anxiety?
17. Overall, how does anxiety affect how you function?
18. How much control do you have over your anxiety?
19. Do you think treatments are effective in treating your anxiety?
20. If there were a pill you could take or a therapy you could do to help with the anxiety, what would you want it to do for you?
21. Has information and support from healthcare services been available, and useful?
22. Have you received any mental health treatment for anxiety, such as talking therapies?
23. Are there any services that have not been useful, or have made things worse?
24. Is there anything else you would like to add?
25. Are there any further questions you think we should add for future interviews?
