# Supplementary material for: The experience of Anxiety for people with Parkinson’s disease
Source: NPJ Parkinsons Dis. 2023 May 17;9:75. doi: 10.1038/s41531-023-00512-1 (PMC10192312; doi:10.1038/s41531-023-00512-1)
Supplement: Supplementary file 2 — Reporting Summary [file 41531_2023_512_MOESM2_ESM.pdf]

nature portfolio

Corresponding author(s): Professor Anette Schrag

Last updated by author(s): 2023/03/13

Reporting Summary

Nature Portfolio wishes to improve the reproducibility of the work that we publish. This form provides structure for consistency and transparency in reporting. For further information on Nature Portfolio policies, see our Editorial Policies and the Editorial Policy Checklist.

Statistics

For all statistical analyses, confirm that the following items are present in the figure legend, table legend, main text, or Methods section.

n/a	Confirmed	
☐	☒	The exact sample size (*n*) for each experimental group/condition, given as a discrete number and unit of measurement
☒	☐	A statement on whether measurements were taken from distinct samples or whether the same sample was measured repeatedly
☒	☐	The statistical test(s) used AND whether they are one- or two-sided<br>*Only common tests should be described solely by name; describe more complex techniques in the Methods section.*
☒	☐	A description of all covariates tested
☒	☐	A description of any assumptions or corrections, such as tests of normality and adjustment for multiple comparisons
☒	☐	A full description of the statistical parameters including central tendency (e.g. means) or other basic estimates (e.g. regression coefficient) AND variation (e.g. standard deviation) or associated estimates of uncertainty (e.g. confidence intervals)
☒	☐	For null hypothesis testing, the test statistic (e.g. *F*, *t*, *r*) with confidence intervals, effect sizes, degrees of freedom and *P* value noted<br>*Give P values as exact values whenever suitable.*
☒	☐	For Bayesian analysis, information on the choice of priors and Markov chain Monte Carlo settings
☒	☐	For hierarchical and complex designs, identification of the appropriate level for tests and full reporting of outcomes
☒	☐	Estimates of effect sizes (e.g. Cohen's *d*, Pearson's *r*), indicating how they were calculated

Our web collection on statistics for biologists contains articles on many of the points above.

Software and code

Policy information about availability of computer code

Data collection	Microsoft Teams, Zoom, REDCap
Data analysis	NVivo 12

For manuscripts utilizing custom algorithms or software that are central to the research but not yet described in published literature, software must be made available to editors and reviewers. We strongly encourage code deposition in a community repository (e.g. GitHub). See the Nature Portfolio guidelines for submitting code & software for further information.

Data

Policy information about availability of data

All manuscripts must include a data availability statement. This statement should provide the following information, where applicable:

- Accession codes, unique identifiers, or web links for publicly available datasets
- A description of any restrictions on data availability
- For clinical datasets or third party data, please ensure that the statement adheres to our policy

The data generated and analysed during the current study are not available as individual privacy could be compromised.

Human research participants

Policy information about studies involving human research participants and Sex and Gender in Research.

Reporting on sex and gender	Our final sample was divided equally by sex to ensure adequate representation of females, following recent guidance from the World Health Organisation and Parkinson's Foundation. Sex was self-reported. Recruitment was ceased at 22 interviews (males = 11) to provide a manageable sample size large enough for adequate comparison between sexes. We found no differences between sexes during analysis, so the results and discussion refer to the dataset as a whole.
Population characteristics	SEE ABOVE
Recruitment	Participants were eligible if in the UK, aged between 18 and 89 years, had a medically confirmed diagnosis of PD, and self-reported anxiety either currently or in the past. Recruitment was done through various channels, including neurology clinics, publicity by the charity Parkinson's UK, a patient and public involvement (PPI) session, and referral by other participants following their interview. Anxiety was not defined in advance of interviews, as we were interested in how participants perceived anxiety and did not wish to lead them in their responses. Taking a self-report approach for anxiety meant we avoided discounting the experiences of those experiencing anxiety who fell below clinically significant thresholds. Our sample therefore includes those who do not meet thresholds as measured by scales, but for whom anxiety is an important part of their subjective experience of PD. This does mean that our results may be affected by recall bias, as participants varied in how long they had experienced anxiety for.
Ethics oversight	University College London Research Ethics Committee

Note that full information on the approval of the study protocol must also be provided in the manuscript.

Field-specific reporting

Please select the one below that is the best fit for your research. If you are not sure, read the appropriate sections before making your selection.

☐ Life sciences ☒ Behavioural & social sciences ☐ Ecological, evolutionary & environmental sciences

For a reference copy of the document with all sections, see nature.com/documents/nr-reporting-summary-flat.pdf

Behavioural & social sciences study design

All studies must disclose on these points even when the disclosure is negative.

Study description	Qualitative
Research sample	People with a confirmed diagnosis of Parkinson's disease (PWP) and self-reported anxiety. We recruited across all disease stages and subgroups with the aim of eliciting diverse perspectives. Interviews were conducted with 22 PWP aged between 43 and 80 years, mean age = 66, 50% female (see Table 1). 20 participants self-reported White British ethnicity, 1 participant self-reported Asian Other, and 1 participant self-reported White-Arab. All self-reported current anxiety. Seven reported taking anxiolytics. GAD-7 scores measuring anxiety ranged from 0 to 19 (clinical cut off > 7) 35, mean score = 6. PAS anxiety scores ranged from 2 to 47 (cut-off > 13)15, mean score = 18. ACE-III scores ranged from 89 to 100 (cut-off for mild cognitive impairment ≤ 89)36, mean score = 96. Two participants reported diagnosed clinical depression at the time of recruitment. The severity of PD was rated using the Hoehn and Yahr scale (scored 0-5; 5 as most severely affected). Participants ranged in their severity of PD, scored as: H&Y score 1 = 7 participants; H&Y score 2 = 2 participants; H&Y score 3 = 13 participants. The study is qualitative and the sample cannot thus be considered statistically representative. The purpose of the study was to explore perceptions of anxiety rather than generalise findings to a population, but we consider the perceptions raised will be prevalent for many PWP. Our sample size was large enough to capture a diverse range of perspectives. However, we note most participants were White British in ethnicity and not many had severe PD according to the H&Y scale. See Table 1 for more participant characteristics.
Sampling strategy	Recruitment was done using a mixture of opportunity and purposive sampling. A sampling matrix ensured our final sample was divided equally by sex to ensure adequate representation of females, following recent guidance from the World Health Organisation and Parkinson's Foundation. We used a maximum variation strategy and recruited across all disease stages and subgroups. Recruitment was ceased at 22 interviews (males = 11) to provide a manageable sample size large enough for adequate comparison between sexes and once data saturation was reached. This sample size is larger than generally considered necessary for scrutinising common perceptions. However, it optimised our ability to collect rich data from different perspectives and is comparable to similar studies.
Data collection	Semi-structured interviews were conducted using video conferencing software (Microsoft Teams and Zoom). All participants chose to have interviews without carers present, so there was no one else present besides the participant and researcher EB. The interview was introduced as an explorative study of anxiety in Parkinson's, but no definition of anxiety was given to avoid leading participants in their responses. Interviews were recorded using Microsoft Teams and Zoom for transcription purposes. All transcripts were anonymised and given unique participant IDs to ensure privacy and compliance with data protection regulation.<br>Consent forms, demographic information, GAD-7 scores and PAS scores were collected using the REDCap online survey platform.

	Participants were emailed a link to complete these surveys. Each participant's responses were saved anonymously using their participant ID.<br>ACE-III scores were collected during assessments for the AND-PD Research Project. These assessments were held remotely on Zoom. No one was present besides the participant and the researcher. The researcher inputted scores into REDCap. Each participant's score was saved anonymously using their participant ID.<br>ACE-III scores were collected during assessments for the AND-PD Research Project. These assessments were held remotely on Zoom. No one was present besides the participant and the researcher. The researcher inputted scores into REDCap. Each participant's score was saved anonymously using their participant ID.
Timing	Data collection start date: 06/01/2022<br>Data collection end-date: 25/04/2022.
Data exclusions	No data excluded from analysis.
Non-participation	No participants dropped out of the study or declined to participate. 3 participants chose not to participate in other parts of the AND-PD study, and thus do not have PAS or ACE-III scores reported.
Randomization	Not applicable.

Reporting for specific materials, systems and methods

We require information from authors about some types of materials, experimental systems and methods used in many studies. Here, indicate whether each material, system or method listed is relevant to your study. If you are not sure if a list item applies to your research, read the appropriate section before selecting a response.

Materials & experimental systems

n/a	Involved in the study
☒	☐ Antibodies
☒	☐ Eukaryotic cell lines
☒	☐ Palaeontology and archaeology
☒	☐ Animals and other organisms
☒	☐ Clinical data
☒	☐ Dual use research of concern

Methods

n/a	Involved in the study
☒	☐ ChIP-seq
☒	☐ Flow cytometry
☒	☐ MRI-based neuroimaging
